# Gaze Characteristics Using a Three-Dimensional Heads-Up Display During Cataract Surgery

**DOI:** 10.3390/jemr18060068

**Published:** 2025-11-17

**Authors:** Puranjay Gupta, Emily Kao, Neil Sheth, Reem Alahmadi, Michael J. Heiferman

**Affiliations:** 1 Department of Ophthalmology and Visual Sciences, Illinois Eye and Ear Infirmary, University of Illinois Chicago, 1855 West Taylor Street, Suite 3.138, Chicago, IL 60612, USA; 2 Department of Ophthalmology, Boston Children’s Hospital, Boston, MA 02115, USA

**Keywords:** eye-tracking, cataract, heads-up display, capsulorhexis, expertise

## Abstract

**Purpose:** An observational study to investigate differences in gaze behaviors across varying expertise levels using a 3D heads-up display (HUD) integrated with eye-tracking was conducted. **Methods:** 25 ophthalmologists (PGY2–4, fellows, attendings; number(n) = 5/group) performed cataract surgery on a SimulEYE model using NGENUITY HUD. **Results:** Surgical proficiency increased with experience, with attendings achieving the highest scores (54.4 ± 0.89). Compared with attendings, PGY2s had longer fixation durations (*p* = 0.042), longer saccades (*p* < 0.0001), and fewer fixations on the HUD (*p* < 0.0001). Capsulorhexis diameter relative to capsule size increased with expertise, with fellows and attendings achieving significantly larger diameters than PGY2s (*p* < 0.0001). Experts maintained smaller tear angles, initiated tears closer to the main wound, and produced more circular morphologies. They rapidly alternated gaze between instruments and surrounding tissue, whereas novices (PGY2–4) fixated primarily on the instrument tip. **Conclusions:** Experts employ a feed-forward visual sampling strategy, allowing perception of instruments and surrounding tissue, minimizing inadvertent damage. Furthermore, attending surgeons maintain smaller tear angles and initiate tears proximally to forceps insertion, which may contribute to more controlled tears. Future integration of eye-tracking technology into surgical training could enhance visual-motor strategies in novices.

## 1. Introduction

Cataract surgery is often recognized as one of the most common surgeries, with more than 26 million cases performed each year [1]. Despite its reputation as a routine and low-complication surgery, successful cataract surgery demands refined psychomotor coordination, visuospatial awareness, and precise control of delicate tissue structures. Even minor deviations in visual attention or manual motion can result in complications such as capsular tears or lens instability, underscoring the importance of visual-motor expertise [2].

In adjacent surgical fields, it has been demonstrated that novice surgeons typically switch their focus between specific points, such as the movement of the surgical instrument and the operative field, reflecting a reactive visual strategy [3]. In contrast, expert surgeons fixate primarily on the operative field to minimize tissue injury, the development of more sophisticated surgical strategies throughout the training process [3]. Traditional cataract surgery training still relies on structured observation, simulation, and graded supervision, yet these methods do not always quantify cognitive or perceptual aspects of expertise [4].

Eye-tracking technology, which maps corneal reflections to track pupillary positions and provides real-time insights into visual attention and cognitive processes during surgery, offers a novel approach to surgical training. Previous research in minimally invasive fields like laparoscopy has demonstrated that experienced surgeons exhibit optimized gaze patterns characterized by enhanced anticipation of instrument trajectories and efficient visual attention distribution on a 2D display [5,6,7,8]. Expert laparoscopic surgeons maintain prolonged fixations on critical anatomical landmarks and demonstrate more systematic scanning, which suggests that eye-tracking technology can reveal advanced visual-motor strategies in experts.

Recent studies have extended this methodology to ophthalmic domains, where head-mounted or microscope-integrated eye tracking can reveal subtle visual differences between trainees and experienced surgeons [9,10,11]. For instance, Nespolo et al. developed a platform for tracking both surgeon and observer gaze in ophthalmic microsurgery, demonstrating the potential for real-time visualization of attention as a surrogate for expertise [9]. However, the integration of 3D heads-up display (HUD) systems with eye-tracking has not yet been investigated, despite their increasing adoption for ergonomics and teaching advantages [12]. In other surgical fields, gaze has been synchronized with hand/tool motion to relate attention to movement smoothness and efficiency; however, gaze-instrument fusion remains underdeveloped in ophthalmic surgery [13]. In the present study, we did not perform gaze-kinematics fusion as the microsurgical environment limited the use of robust external tracking. Instead, we quantify HUD-specific gaze behaviors and link them to surgical descriptors.

Cataract surgery is our chosen model for applying and evaluating eye-tracking technology due to its high prevalence, standardized procedure, and critical need for visual attention and precision [14]. Furthermore, utilizing a 3D HUD integrated with eye-tracking technology can provide greater detail about where experts fixate during surgery. By analyzing the gaze dynamics of surgeons in conjunction with surgical techniques, we aim to identify specific strategies applicable to the use of 3D HUDs in ophthalmic surgery that can be imparted to trainees, providing immediate, actionable feedback and facilitating targeted educational programs to facilitate the acquisition of surgical skills and advance ophthalmic surgical education.

## 2. Materials and Methods

We conducted an Institutional Review Board-approved, observational study involving ophthalmology trainees and faculty at the Illinois Eye and Ear Infirmary, University of Illinois at Chicago. Participants were recruited across various levels of experience, including postgraduate year 2 (PGY2), year 3 (PGY3), and year 4 (PGY4) ophthalmology residents, anterior segment and vitreoretinal surgery fellows, and attending cataract surgeons. A total of 25 participants were included, with 5 individuals in each group (PGY2, PGY3, PGY4, fellows, and attendings). All subjects were selected based on availability and willingness to participate and had normal or corrected-to-normal vision (20/20 or better). Participant demographics are characterized in Table 1.

Participants performed multiple steps of cataract surgery on a SimulEYE model (Westlake Village, CA, USA) using an NGENUITY (Alcon, Fort Worth, TX, USA) heads-up display. The task required participants to remove a red film from the model, utilizing the same instruments (cystotome and Utrata forceps) and steps required for a continuous curvilinear capsulorhexis (CCC). Specific tasks included tri-planar corneal wound creation, creation of a CCC, and wound closure with a single interrupted 10-0 nylon suture.

The participants were prepared using protocols derived from studies utilizing eye-tracking systems for visual diagnostic performance assessment [15,16]. The experimental setup consisted of a standard operating room environment with the SimulEYE model and the NGENUITY heads-up display. Participants controlled the pace of their procedures, and the duration spent on each task was recorded. A verbal prompt confirmed the proper calibration of the eye-tracking device before the start of the procedure. A power analysis was conducted prior to the study, using effect size estimates obtained from previous gaze-tracking studies [17]. Specifically, earlier findings indicated a Cohen’s f of 1.36 for comparisons across three levels of experience. Inputting this value into a one-way ANOVA power calculation (α = 0.05, 1 − β = 0.80, k = 3) suggested that a minimum of three participants per group would be sufficient. However, to account for potentially higher variability in cataract procedures and to align with established standards in gaze-tracking research, we recruited 5 individuals for each training group.

The mobile eye-tracking device used in this study was the Pupil Core eye-tracker (Pupil Labs, Berlin, Germany). The Pupil Core eye-tracker features three cameras: two infrared cameras that monitor the eyes at 200 frames per second (FPS) with a resolution of 192 × 192 pixels and an outward-facing visible light camera that records at 30 FPS with a 1280 × 720 pixel resolution and a diagonal field of view (FOV) of 100°.

Eye-tracking recordings were analyzed based on several parameters, focusing on fixation and saccades. Fixations, defined as periods during which the eye remains relatively stationary, were classified with a maximum dispersion of 1.50 degrees and a duration range of 80 to 420 milliseconds. Saccades, the rapid movements of the eyes between fixations, were also analyzed. Quantitative metrics included the number of fixations, fixation durations, blink rates, saccade length, and time distribution between the heads-up display/area of interest, the surgical field, and other areas. The distribution of fixations across these locations and the length of the tear during the capsulorhexis were also recorded.

The area of the capsulorhexis model fixated on during surgery was determined using an image processing algorithm within the Pupil Core tracker system. SIMULEYE model images were labeled with a baseline blue mask, and a 5° circular marker was applied at each point of the eye’s x, y, and z positions during the trial, creating a non-blue mask over the baseline image. Coverage was quantified by comparing the non-blue pixels (representing covered areas) in the post-trial image to those in the original image. Qualitative data, including gaze patterns, were compared between groups and related to existing literature. Surgical ratings were assigned from the recordings using a modified International Council of Ophthalmology-Ophthalmology Surgical Competency Assessment Rubric-Phacoemulsification (ICO-OSCAR; Supplementary Table S1). These ratings were assigned by two blinded authors who viewed the recording after collection, and the average score between the authors was used.

Descriptive statistics are reported in the table below. Parametric tests were selected as the aggregated gaze metrics approximated normal distributions when averaged across several hundred data points per participant. Correlation analyses were performed using the Pearson formula in Origin(Pro), Version Number 2024 (OriginLab Corporation, Northampton, MA, USA). A five-group (PGY2, PGY3, PGY4, Fellow, and Attending) analysis of variance (ANOVA) was performed with a post hoc Tukey’s test (n = 5 for all participant groups; 5 groups; α = 0.05). *p* ˂ 0.05 was considered statistically significant.

## 3. Results

### 3.1. Surgical Outcomes

**Figure 1:**
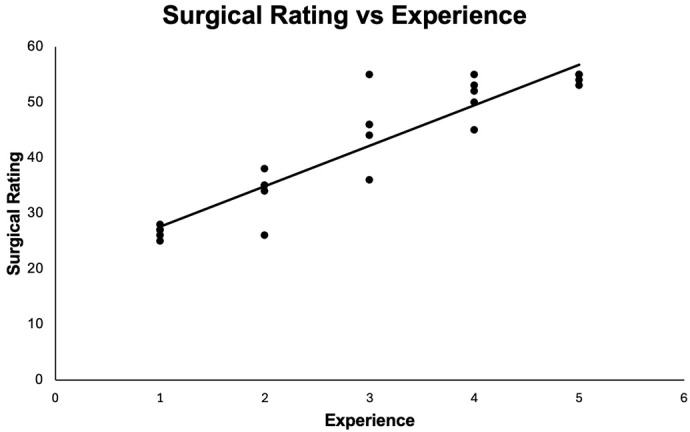
Pearson correlation graph correlating level of expertise and surgical rating. Surgical rating was assessed using a modified ICO-OSCAR rubric. Experience was assigned an ordinal variable where PGY2—1, PGY3—2, PGY4—3, fellow—4, and attending—5. Strong linear correlation supported (R^2^ = 0.927, N = 25, *p* < 0.0001).

Surgical proficiency was assessed by a modified ICO-OSCAR score to analyze the correlation between expertise and surgical rating (Table 2). Proficiency scores increased with experience, demonstrating a strong positive correlation between training level and surgical aptitude (R^2^ = 0.93, *p* < 0.0001) (Figure 1). In general, PGY2s exhibited the lowest level of proficiency with the fellows and attendings obtaining the highest average proficiency scores.

### 3.2. Fixations

**Figure 2:**
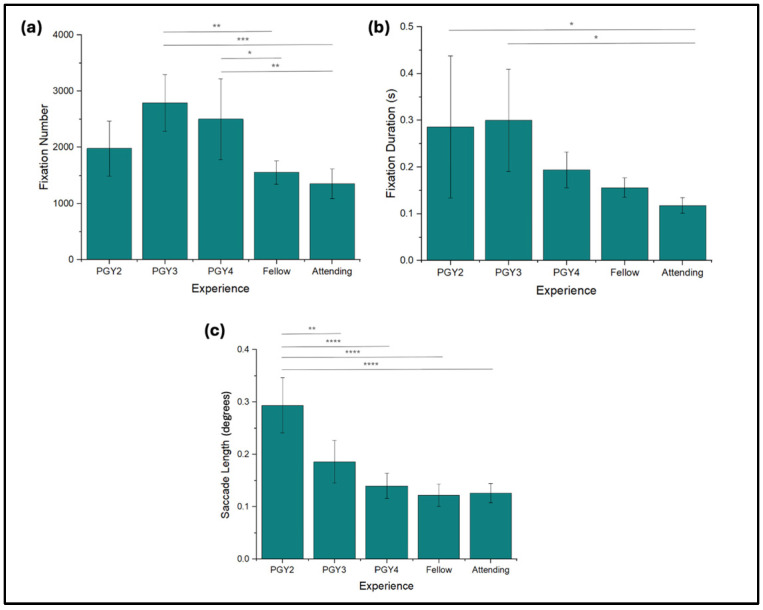
Gaze characteristics data among expertise levels. Fixations are defined as groupings of consecutive points within 1.50 degrees of dispersion and above the confidence threshold (**a**) fixation number. (**b**) fixation duration (s) within the range of 80 to 420 milliseconds. (**c**) length of saccades, or shifts to the center of gaze, measured by degrees of dispersion. * *p* < 0.05, ** *p* < 0.01, *** *p* < 0.001, **** *p* < 0.0001.

Fixation numbers demonstrated a general reduction as experience increased. PGY3s exhibited the highest average fixation number at 2791 ± 503 (Figure 2a), while the attendings recorded significantly shorter fixation numbers at 1355 ± 261 (*p* < 0.0009). Fixation duration also decreased with experience. PGY2s displayed the highest average fixation duration at 0.286 ± 0.152 s (Figure 2b), and attendings again exhibited significantly lower duration of 0.118 ± 0.016 s (*p* = 0.042).

### 3.3. Saccade Length

The next metric analyzed was saccade length, which was reduced as the level of training increased from PGY2s to fellows (Figure 2c). PGY2s exhibited the longest saccades with an average length of 0.294 ± 0.053° of dispersion, significantly shorter than PGY4s (*p* < 0.001). The saccade lengths of fellows and attendings continued to decrease but were not significantly different from one another, with lengths of 0.122 ± 0.022° and 0.126 ± 0.018°, respectively.

### 3.4. Gaze Distribution

**Figure 3:**
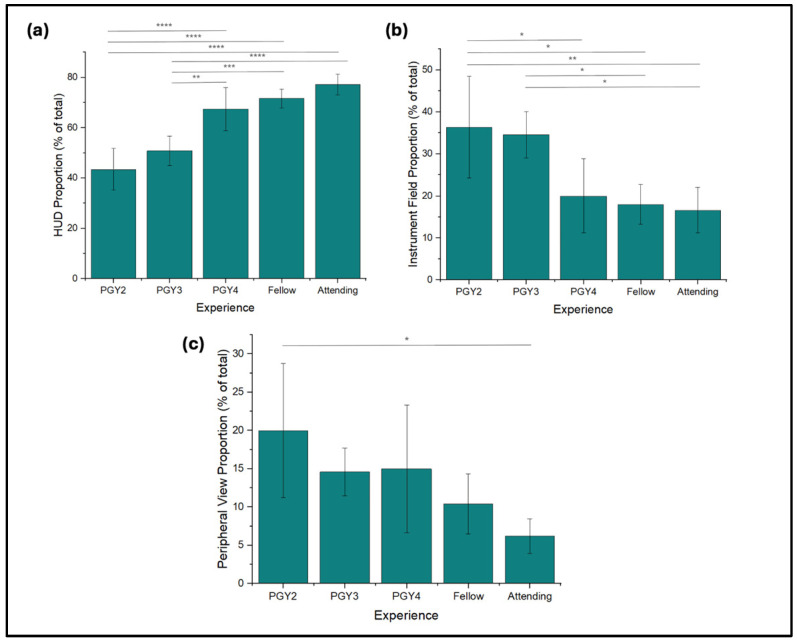
Gaze distribution data among expertise levels. Percentages were determined as the proportion of total fixations that fell within the defined areas. Areas were partitioned via surface tracking. (**a**) HUD proportion. (**b**) Instrument field, or the area of the SimulEYE model, proportion. (**c**) Peripheral View, or areas outside immediate surgical relevance, proportion. * *p* < 0.05, ** *p* < 0.01, *** *p* < 0.001, **** *p* < 0.0001.

By leveraging surface tracking markers, we collected information on the distribution of fixations within each area of the surgical environment and again analyzed the relationship with different levels of expertise (Figure 3a–c). The most considerable trend was an observed increase in the proportion of total fixations allocated to the HUD with experience. Attendings spent an average of 77.2 ± 4.09% of fixations on the heads-up display, allotting significant focus to the device relative to PGY2s (*p* < 0.0001). PGY2s, on the other hand, allocated an average of 43.4 ± 8.32% to the HUD. Correspondingly, attendings spent the least number of fixations dedicated to the instrument field, at 16.6 ± 5.5%, and PGY2s appeared to spend more at 36.4 ± 12.1%.

### 3.5. Capsulorhexis Diameter

**Figure 4:**
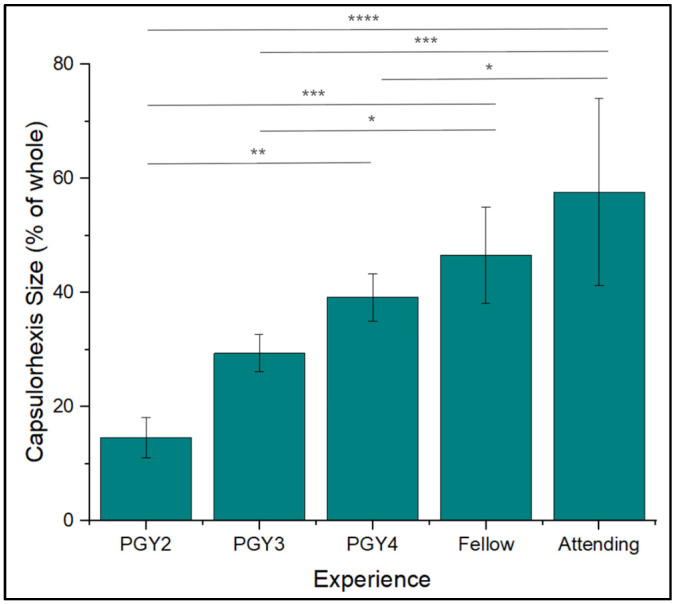
Capsulorhexis size data among expertise levels. Percentages were determined as the length of the tear created during capsulorhexis over the length of the full capsulorhexis model. * *p* < 0.05, ** *p* < 0.01, *** *p* < 0.001, **** *p* < 0.0001.

Capsulorhexis diameter was another metric that appeared to increase with medical training (Figure 4). PGY2s created tears with diameters that averaged 14.6 ± 3.5% of the lens capsule, which was smaller than PGY3s (*p* < 0.0001). Fellows and attendings averaged tears of 46.6 ± 8.44% and 57.6 ± 16.4%, respectively, the largest proportions among groups. Overall, it was observed that attendings and fellows created a capsulorhexis with a larger diameter during the cataract surgery process.

### 3.6. Area of Fixation

**Figure 5:**
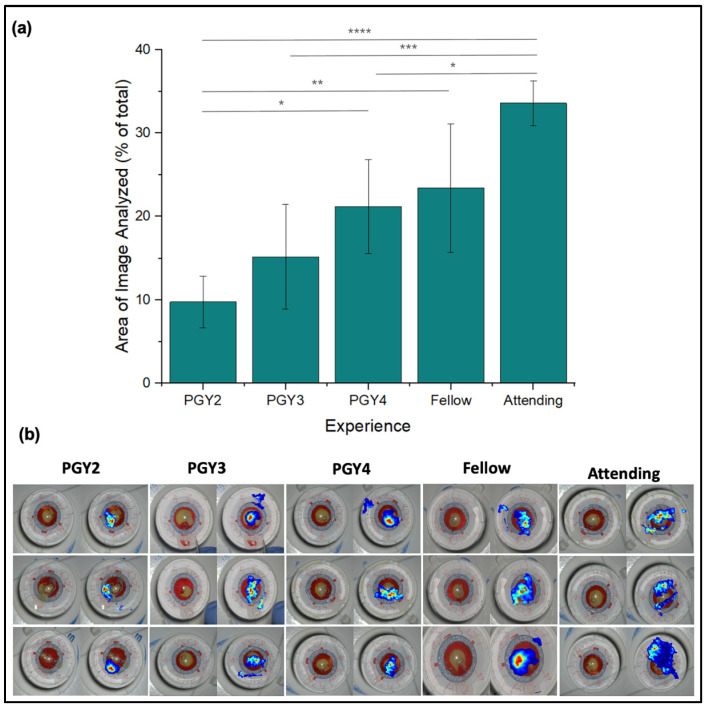
Gaze area among expertise levels. (**a**) Area of the instrument field fixated upon as determined by the number of non-blue pixels recorded by fixation markers over the total number of pixels in the baseline image. Image processing was carried out via ImageJ (1.54r). (**b**) Gaze heat map data overlayed on top of the completed surgical landscape. Red areas represent areas of high fixation, while blue areas represent areas of lower fixation. Colorless areas represent areas of minimal fixation. Rows contain data from the three participants of each expertise level in no particular order. * *p* < 0.05, ** *p* < 0.01, *** *p* < 0.001, **** *p* < 0.0001.

Our analysis continued with the percentage of the instrument field fixated upon by each expertise level. PGY2s fixated on the narrowest portion of the instrument field, averaging 9.8 ± 5.19%, significantly smaller than attendings (*p* < 0.0001) (Figure 5a). The area of fixation increased slightly with each level of training before increasing substantially to a maximum of 33.6 ± 2.7% of the field fixated upon by the attendings. Additionally, fixations by expert surgeons were more evenly distributed over a broad expanse of the capsulorhexis model, covering areas unaltered by surgical intervention (Figure 5b). Novice (PGY2-PGY4) fixation density was notably highest at the tear area and limited at the adjacent surroundings.

### 3.7. Angle of Tear Relative to Forceps

**Figure 6:**
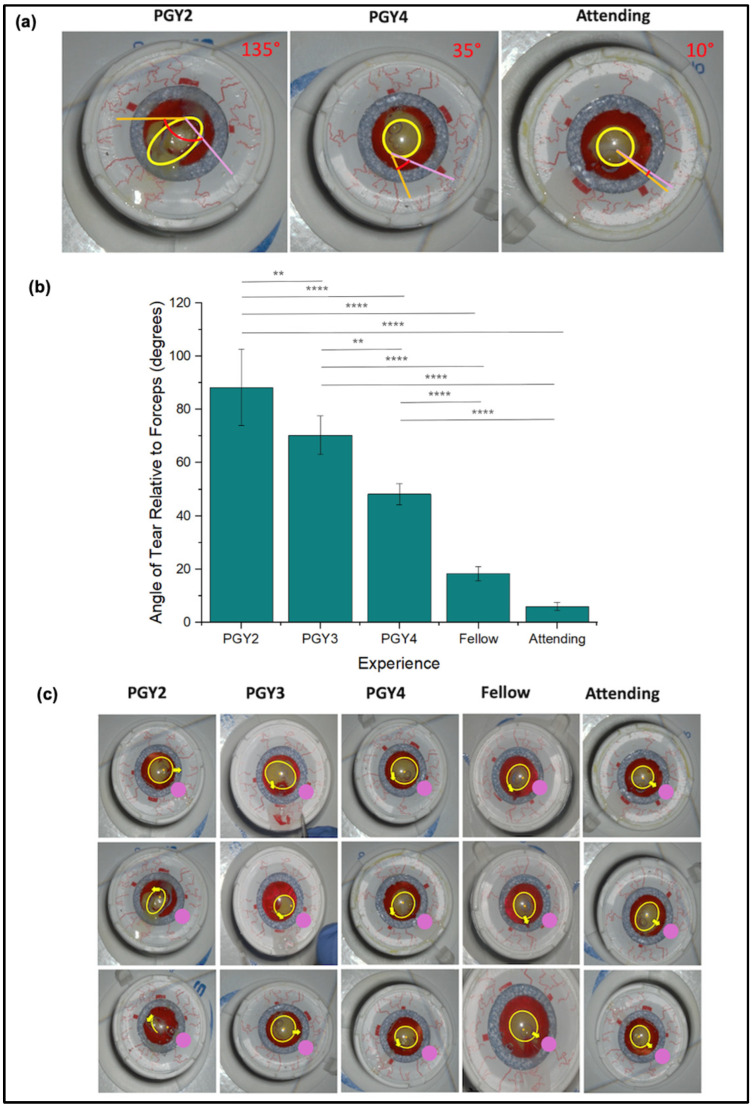
Capsulorhexis Tear Morphology Across Experience Levels. (**a**) Representative capsulorhexis tears from three surgeons at varying experience levels. The pink line indicates the forceps’ position during the tear; the orange line represents the tear vector; the yellow circle traces the forceps’ path; and the red angle denotes the measured angle between the tear vector and forceps position. Estimated angles for each sample are displayed in red in the top right corner. (**b**) Angle between the tear vector and forceps position among expertise levels. Angles were derived using the analysis demonstrated in the representative tears. (**c**) Capsulorhexis tears from all participants. The yellow circles depict the forceps’ travel paths; yellow arrows indicate the tear vectors and initiation points; and pink lines show the forceps’ insertion points into the model. ** *p* < 0.01, **** *p* < 0.0001.

Expertise also demonstrated an inverse relationship with the angle of tear relative to the forceps, as demonstrated in the sample data in Figure 6a,b. Specifically, PGY2 residents demonstrated the largest angle, 88.4° ± 14.3°, followed by PGY3 at 70.4° ± 7.30°, PGY4 at 48.4° ± 4.03°, Fellows at 18.4° ± 2.7°, and Attendings, who maintained the smallest angle of 6.0° ± 1.58°. Statistical analysis showed significant differences across levels of expertise. Notably, PGY2 residents exhibited significantly higher angles than PGY4 (*p* < 0.0001), fellow (*p* < 0.0001), and attending groups (*p* < 0.0001). PGY3 residents also demonstrated significantly greater angles than fellows (*p* < 0.0001) and attendings (*p* < 0.0001).

### 3.8. Tear Morphology

Analysis of tear morphology across expertise levels (Figure 6c) reveals distinct differences in technique between attendings and novice groups. Attendings demonstrate a controlled, radial approach, creating nearly circular capsulorhexis tears by pulling outward in a uniform direction relative to the circle’s center. By pivoting the forceps in the wound, a more consistent, complete circular tear profile is created. In contrast, novice groups display a more tangential approach, pulling in directions aimed toward the exterior of the intended circular path, leading to irregular and asymmetric tear shapes.

Additionally, the initiation point of the tear differs across expertise levels. Attendings tend to begin the tear closer to the forceps insertion site, indicating a refined control over initial incision placement. Novice groups, however, initiate the tear from positions farther from the forceps, resulting in less precision and control over the tear path.

### 3.9. Qualitative Observations

Recordings of each surgical procedure were also analyzed qualitatively according to the established literature. Of note, during the main incision step and, more significantly, during the CCC, more experienced surgeons exhibited rapid shifts in their visual focus between the instrument tip and the surrounding tissues. This contrasts with novice surgeons, who primarily concentrate their gaze on the instrument tip and its immediate vicinity.

## 4. Discussion

Understanding the spatiotemporal traits of eye-hand coordination in surgery may be used to enhance surgical training, particularly in ophthalmology. This study investigated whether experienced surgeons exhibit distinct gaze behaviors and surgical techniques that correlate with their superior performance during simulated cataract surgery. The four major metrics assessed included gaze characteristics (fixation number, fixation duration, and saccade length), gaze distribution (across the HUD, surgical field, and the instrument), tear morphology, and surrounding tissue monitoring. The findings reveal that expert surgeons exhibited shorter fixation durations, a more focused gaze distribution on the HUD, longer continuous capsulorhexis tears with a more proximal tear initiation, and a smaller angle of tear relative to the forceps, as well as gaze distribution beyond the flap interface, indicating efficient visual processing and anticipation of surgical actions.

Previous studies have established that significant increases in fixation durations correlate with mental workload and task complexity [7,18,19,20]. In this study, we observed that expert surgeons exhibited significantly shorter fixation durations and saccade lengths. The lack of significantly higher fixation numbers among PGY2s likely reflects shorter operative times resulting from inexperience rather than a true deviation in gaze behavior. These trends suggest an improved ability to integrate visual information efficiently and a reduced cognitive load during surgery. This interpretation is supported by Zheng et al., who noted that surgical trainees often report lower confidence during laparoscopy, correlating with increased fixation times and mental workload [21]. Similar findings have been demonstrated in high-fidelity microsurgical simulations, where gaze entropy and fixation clustering metrics effectively discriminated between expert and novice performance, suggesting that gaze-based markers may serve as reliable indicators of visuomotor efficiency [15,22]. This ability to swiftly process visual information may be a key factor that distinguishes expert performance and contributes to improved surgical precision, as demonstrated by experts’ significant increase in ICO-OSCAR score, and suggests that this can be an area of focus for training programs to improve trainee performance.

The distribution of visual attention as measured by the proportion of time spent looking at the HUD versus other areas of the surgical field was also analyzed. Previous research, including a study by Liu et al., revealed that experts in laparoscopic surgery focus more on areas of interest that capture future surgical targets within the 2D display environment [5,8,23]. Liu et al. attributed these observations to a “feed-forward” visual sampling approach, where experts will gaze at upcoming surgical targets and rapidly bounce back to their tools. This trend was exhibited in our data in which more experienced surgeons spent a significantly greater proportion of their gaze on the HUD, while novices appeared to spend a greater time in the surgical field and elsewhere. The findings from our study are corroborated by the Nespolo et al. study, which determined that resident ophthalmic surgeons tend to focus more on the instrument tooltip than fellows and attendings, indicating decreased anticipation and preparation for subsequent surgical actions [9]. The present findings thus extend prior evidence of predictive gaze to the 3D HUD environment.

Regarding surgical technique, tear morphology and capsulorhexis performance metrics in this study further illustrate how refined visuomotor control can translate to tangible surgical approaches. CCC diameter increased with higher levels of expertise, suggesting a smoother execution with lower jerk measurements—a quantitative indicator of motor control that novices typically lack. Prior studies have demonstrated that motion smoothness correlates strongly with expertise and task efficiency across various surgical domains [24,25]. The CCC diameter was evaluated as part of the surgical assessment score, and no cases exceeded the threshold of 6–6.5 mm in diameter, beyond which the intraocular lens optic would risk improper seating and potential displacement.

Additionally, it was observed that attending surgeons consistently achieve smaller tear angles relative to the forceps, initiate tears proximal to the insertion site, and produce more circular and regular tear morphologies. The initiation of tears closer to the main wound by attending surgeons may reflect a learned approach to maintaining control over the direction and size of the tear, indicating internalized eye-hand coordination strategies developed through experience [26,27]. This data also shows that the attendings are pivoting the forceps in the wound, which reinforces one of the lessons taught to trainees about creating a good capsulorhexis. This technique appears to reduce the risk of radial extensions and creates a more circular tear.

Furthermore, the extent of the operative field visualized by surgeons correlated positively with their level of expertise. Attending surgeons, who visually cover a larger portion of the operative field, likely benefit from a more comprehensive understanding of the surgical environment, thereby enhancing their situational awareness and decision-making capabilities during procedures. The larger operative field awareness also likely decreases damage to the surrounding anterior capsule during cataract surgery amongst attendings compared to trainees, which has been well documented in the literature [28,29,30]. As such, the improved visualization strategies, coupled with surgical techniques that facilitate the creation of controlled tears, may contribute to expert performance in surgery.

The present findings suggest that surgeons who exhibit more developed gaze behaviors characterized by shorter fixation durations, efficient gaze transitions, and broader operative field coverage also tend to demonstrate superior surgical techniques, such as smoother tear morphology and controlled capsulorhexis formation. Although our study did not directly correlate eye-tracking metrics with hand kinematics, these results imply that refined visual strategies likely support improved motor control. Recent advances in multimodal surgical analytics have demonstrated that coupling gaze metrics with motion tracking and force data can further enhance the precision of expertise modeling [31]. Incorporating these multimodal data streams into ophthalmic surgical education may allow for more nuanced assessment and targeted feedback beyond traditional video-based review. Similarly, modern machine learning models trained on gaze and kinematic data have achieved high accuracy in classifying surgeon expertise across multiple surgical tasks [32,33], suggesting potential applications for objective skill assessment and personalized feedback in cataract surgery training.

Although cataract surgery is often described as a “routine” or standardized procedure, these findings highlight that expert performance relies on complex and highly refined visuomotor coordination. Eye-tracking analysis provides objective evidence that successful cataract surgery demands sophisticated, non-trivial perceptual and cognitive skills developed through extensive experience. Recognizing this complexity reframes cataract surgery as a dynamic process requiring advanced spatial awareness and predictive control. This perspective reinforces the importance of structured simulation-based training and the continued investigation of gaze-based metrics as tools for assessing and improving surgical expertise.

While the findings of this study are significant, we acknowledge several limitations. The study’s sample size was small and within a single institution, which may limit the generalizability of the results. Additionally, while performance in simulated cataract surgery as well as with eye-tracking glasses has been independently validated to correlate with real-world performance in similar models, the specific simulation setup utilized has yet to undergo external validation [6,34]. Furthermore, all participants in each respective resident group were sampled from the same training year. However, assessing the surgical ability of the fellows and attending surgeons yields further variability in results due to differences in the quality of residency and other post-graduate training. It is also possible that the observed differences reflect varying degrees of familiarity with the HUD system rather than true differences in surgical expertise. Given the inherently non-standard differences in experience among fellows and attending surgeons, we have attempted to control for all known tangible confounding variables. Furthermore, while surgical simulation provides a controlled and reproducible environment, its ability to predict real-world operative outcomes remains inherently limited. Lastly, the reliance on eye-tracking technology introduces potential biases, such as calibration errors or variability in individual gaze patterns, that could affect the accuracy of the measurements. These factors might skew the results, particularly in detecting subtle differences in gaze behaviors. Future research with larger sample sizes, integration of real surgical data, and advanced computational modeling could address these limitations and further validate the findings.

## 5. Conclusions

Despite these limitations, the present findings have important implications for ophthalmology surgical education, particularly in incorporating HUD technology coupled with eye-tracking in training programs. The above evidence suggests that this technology could help bridge the expertise gap in terms of manual skills and in developing efficient gaze behaviors. Previous research indicates that exposing trainees to expert gaze behaviors, either through pre-recorded or real-time HUDs, can accelerate learning and enhance performance by allowing trainees to visualize and emulate the “gaze template” used by experts [35,36,37]. Furthermore, eye-tracking measures are proven to demonstrate greater sensitivity to surgical expertise than behavioral measures or surgical outcomes and may be explained by the greater sensitivity of eye-tracking that can measure dozens/hundreds of fixations per task [38,39].

This study highlights differences in the spatiotemporal traits of eye-hand coordination between expert and novice surgeons using eye-tracking and a 3D HUD. These differences are significant as they provide evidence for the sophisticated, non-trivial skills required for expert cataract surgery performance, as well as insights into the visual strategies that inform this surgical proficiency. When considered alongside differences in surgical technique, our results indicate that mature gaze control may support the precise motor execution required for optimal surgical outcomes [40]. While our current analysis focused on gaze behavior and surgical approach separately, extending previous studies’ coupling of eye-tracking with motion kinematics to cataract surgery may reveal how gaze and hand movements co-evolve through training and identify the perceptual–motor features most predictive of surgical success [13,41]. Future studies should therefore integrate synchronized eye- and hand-tracking data, ideally within 3D heads-up display (HUD) platforms, to capture the full spectrum of surgical expertise. Doing so will enable more precise and individualized feedback for trainees, helping to establish objective and scalable benchmarks for surgical proficiency. Bridging eye-movement patterns with intraoperative performance metrics may pave the way toward automated, real-time assessment tools that enhance both training and patient safety in ophthalmic surgery [42].

## Figures and Tables

**Table 1:** Participant Demographic Data. Demographics of participants, including age, years of experience starting from PGY2, and number of males vs. females.

Current Position	PGY2	PGY3	PGY4	Fellows	Attendings
Sample (n)	5	5	5	5	5
Age (yrs; mean ± sd)	29.3 ± 1.4	30.2 ± 1.21	31.5 ± 2.12	33.1 ± 2.54	44.5 ± 8.32
Years of Experience	0.5 ± 0.13	1.4 ± 0.23	2.2 ± 0.42	4.4 ± 0.72	14.5 ± 3.61
Males/Females	3/2	2/3	3/2	4/1	2/3

**Table 2:** Experience and Surgical Scores. Prior experience and competency score by current position collected via post-performance surveys. This includes the average number of previously performed relevant procedures and the modified ICO-OSCAR score received during the recorded surgical procedure.

Current Position	PGY2	PGY3	PGY4	Fellows	Attendings
Sample (n)	5	5	5	5	5
No. of Capsulorhexis Procedures	0 ± 0	15 ± 1.31	115 ± 14.12	292.9 ± 85.41	478.31 ± 121.48
No. of Corneal	0.6 ± 0.91	12 ± 1.11	123 ± 21.71	345 ± 132.13	632 ± 292.22
No. of Wound Closure	0 ± 0	7.37 ± 4.69	56 ± 32.53	176 ± 224.97	363 ± 287.09
Modified ICO-OSCAR Score	26 ± 1.14	33.6 ± 4.51	45.4 ± 6.76	51 ± 3.81	54.4 ± 0.89

## Data Availability

The original contributions presented in this study are included in the article/Supplementary Materials. Further inquiries can be directed to the corresponding author.

## Supplementary Materials

The following supporting information can be downloaded at: https://www.mdpi.com/article/10.3390/jemr18060068/s1, Table S1: ICO-Ophthalmology Surgical Competency Assessment Rubric-Phacoemulsification. This Is Attached In The Zip File.

## Abbreviations

The following abbreviations are used in this manuscript:

HUD	Heads-up display
CCC	Continuous curvilinear capsulorhexis
