# Supplementary material for: Gaze Characteristics Using a Three-Dimensional Heads-Up Display During Cataract Surgery
Source: J Eye Mov Res. 2025 Nov 17;18(6):68. doi: 10.3390/jemr18060068 (PMC12641938; doi:10.3390/jemr18060068)
Supplement: Supplementary file 1 [file jemr-18-00068-s001.zip › jemr-3898260-supplementary.pdf]

ICO-Ophthalmology Surgical Competency Assessment Rubric-Phacoemulsification (ICO-OSCAR:phaco)					
	**Novice (score = 2)**	**Beginner (score = 3)**	**Advanced Beginner (score = 4)**	**Competent (score = 5)**	**Score**
**Incision & Paracentesis: Formation & Technique**	Inappropriate incision architecture, location, and size.	Leakage and/or iris prolapse with local pressure, provides poor surgical access to and visibility of capsule.	Incision either well-placed or non-leaking but not both.	Incision parallel to iris, self sealing, adequate size, provides good access for surgical maneuvering.	
**Viscoelastic: Appropriate Use and Safe Insertion**	Unsure of when, what type and how much viscoelastic to use. Has difficulty accessing anterior chamber through paracentesis.	Requires minimal instruction. Knows when to use but administers incorrect amount or type.	Requires no instruction. Uses at appropriate time. Administers adequate amount and type. Cannula tip in good position. Unsure of correct viscoelastic if multiple types available.	Viscoelastics are administered in appropriate amount and at the appropriate time with cannula tip clear of lens capsule and endothelium. Appropriate viscoelastic is used if multiple types of viscoelastics are available.	
**Capsulorrhexis: Commencement of Flap & followthrough.**	Instruction required, tentative, chases rather than controls rhexis, cortex disruption may occur.	Minimal instruction, predominantly in control with occasional loss of control of rhexis, cortex	In control, few awkward or repositioning movements, no cortex disruption.	Delicate approach and confident control of the rhexis, no cortex disruption.	

		disruption may occur.			
**Capsulorrhexis: Formation and Circular Completion**	Size and position are inadequate for nucleus density & type of implant, tear may occur.	Size and position are barely adequate for nucleus density and implant type, difficulty achieving circular rhexis, tear may occur.	Size and position are almost exact for nucleus density and implant type, shows control, requires only minimal instruction.	Adequate size and position for nucleus density & type of implant, no tears, rapid, unaided control of radialization, maintains control of the flap and AC depth throughout the capsulorrhexis.	
**Wound Closure (Including Suturing, Hydration, and Checking Security as Required)**	If suturing is needed, instruction is required and stitches are placed in an awkward, slow fashion with much difficulty, astigmatism, bent needles, incomplete suture rotation and wound leakage may result, unable to remove viscoelastics thoroughly. unable to make incision watertight or does not check wound	If suturing is needed, stitches are placed with some difficulty, resuturing may be needed, questionable wound closure with probable astigmatism, instruction may be needed, questionable whether all viscoelastics are thoroughly removed, Extra maneuvers are required to make the incision watertight at the end of	If suturing is needed, stitches are placed with minimal difficulty tight enough to maintain the wound closed, may have slight astigmatism, viscoelastics are adequately removed after this step with some difficulty, The incision is checked and is water tight or needs minimal adjustment at the end of the	If suturing is needed, stitches are placed tight enough to maintain the wound closed, but not too tight as to induce astigmatism, viscoelastics are thoroughly removed after this step, the incision is checked and is water tight at the end of the surgery. Proper final IOP.	

	for seal. Improper final IOP.	the surgery. May have improper IOP.	surgery. May have improper IOP.		
**Global Indices**					
**Wound Neutrality and Minimizing Eye Rolling and Corneal Distortion**	Nearly constant eye movement and corneal distortion.	Eye often not in primary position, frequent distortion folds.	Eye usually in primary position, mild corneal distortion folds occur.	The eye is kept in primary position during the surgery. No distortion folds are produced. The length and location of incisions prevents distortion of the cornea.	
**Eye Positioned Centrally Within Microscope View**	Constantly requires repositioning.	Occasional repositioning required.	Mild fluctuation in pupil position.	The pupil is kept centered during the surgery.	
**Conjunctival and Corneal Tissue Handling**	Tissue handling is rough, and damage occurs.	Tissue handling borderline, minimal damage occurs.	Tissue handling decent but potential for damage exists.	Tissue is not damaged nor at risk by handling.	
**Intraocular Spatial Awareness**	instruments often in contact with capsule, iris and corneal endothelium', blunt second hand instrument not kept in appropriate position.	Occasional accidental contact with capsule, iris and corneal endothelium, sometimes has blunt secondhand instrument between the posterior capsule and the activated phaco tip.	Rare accidental contact with capsule, iris and corneal endothelium. Often has blunt secondhand instrument between the posterior capsule and the activated phaco tip.	No accidental contact with capsule, iris and corneal endothelium, when appropriate, a blunt, secondhand instrument, is always kept between the posterior capsule and	

				the tip of the phaco when the phaco is activated.	
**Iris Protection**	Iris constantly at risk, handled roughly.	Iris occasionally at risk. Needs help in deciding when and how to use hooks, ring or other methods of iris protection.	Iris generally well protected. Slight difficulty with iris hooks, ring, or other methods of iris protection.	Iris is uninjured. Iris hooks, ring, or other methods are used as needed to protect the iris.	
**Overall Speed and Fluidity of Procedure**	Hesitant, frequent starts and stops, not at all fluid.	Occasional starts and stops, inefficient and unnecessary manipulations common, case duration about 60 minutes.	Occasional inefficient and/or unnecessary manipulations occur, case duration about 45 minutes.	Inefficient and/or unnecessary manipulations are avoided, case duration is appropriate for case difficulty. In general, 30 minutes should be adequate.	
**Final Score: ____/55**					
